# Peer‐supported faculty development and workplace teaching: an integrative review

**DOI:** 10.1111/medu.13896

**Published:** 2019-06-25

**Authors:** Narelle Campbell, Helen Wozniak, Robyn L Philip, Raechel A Damarell

**Affiliations:** ^1^ Flinders NT, College of Medicine and Public Health, Flinders University Darwin Northern Territory Australia; ^2^ Prideaux Centre for Research in Health Professions Education College of Medicine & Public Health Bedford Park South Australia Australia; ^3^ Office of Medical Education, Faculty of Medicine University of Queensland Brisbane Queensland Australia; ^4^ College of Medicine & Public Health Bedford Park South Australia Australia

## Abstract

**Context:**

The use of peer support as a faculty development technique to improve clinical teaching is uncommon in medical education, despite the benefits of situating learning in the workplace. The authors therefore conducted a broad search seeking theoretical and empirical literature describing peer support strategies for clinical teachers in health care workplaces. This included descriptive and non‐experimental studies that are often excluded from reviews. The review aimed to identify and assess existing initiatives and to synthesise key challenges and benefits.

**Methods:**

An integrative literature review was undertaken (2004–2017), based on searches of eight international electronic databases and targeted manual searches. Key concepts, elements and models were mapped using an iterative, constant comparative method. An evaluative framework, drawing on previous research, informed conclusions regarding the quality of evidence.

**Results:**

From a pool of 5735 papers, 34 met the inclusion criteria. The majority referred to studies conducted in the USA (59%) and in the medical profession (71%). Analysis revealed a trend towards using a collaborative model (56%), voluntary participation (59%), and direct workplace observation by a peer clinician (68%). Design features of the peer support strategy were commonly reported (65%), with half providing outcome measures (56%). Few papers reported on process evaluation (15%) or evidence of programme sustainability (15%). Despite logistical and time‐associated challenges, benefits accrued to individuals and the workplace, and included improved teaching practices. Embedding the peer support strategy into routine organisational practice proved effective.

**Conclusions:**

The results indicated that a workplace‐based peer support model is an acceptable and effective faculty development strategy for health care clinical teachers. Conceptualising workplace‐based peer support via a sociocultural model that acknowledges the significance of educational design, peers as collaborators and the importance of workplace context and culture is emphasised. Future research should focus on clarification studies informed by contemporary models of faculty development, in which factors impacting the health care workplace are considered.

## Abstract 2

By reviewing the literature, Campbell et al. highlight how collaborative relationships and workplace connections with peers offer an under‐utilized resource for effectively improving teaching expertise.

## Introduction

Practising clinicians provide a significant amount of workplace‐based teaching and supervision for students and early career health professionals. The importance of the clinical teacher role in providing the learning nexus between the patient,^1^ application of knowledge,^2^ role identity development^3^ and learner cannot be overstated. Many clinicians teach, with little or no training, because it is an expected part of their work portfolio. Classroom‐based, workshop‐style faculty development, attended by individuals, may provide theoretical understanding, but cannot ensure transfer of skills and knowledge into the workplace and workplace culture.^4^ Accessible and effective faculty development to expand clinician teaching expertise is critical^5^ as a means of moving beyond the apprenticeship model^6^, ^7^ so often found in workplace‐based medical education.

One option for improving clinical teaching effectiveness in the clinical workplace is to develop strategies that foster connections and relationships between trusted colleagues. By adopting the role of peer mentor,^5^, ^8^, ^9^ a colleague can assist a peer to develop his or her skills of critical reflection on teaching practice. As models of faculty development evolve, the importance of relationships between facilitators, participants and professional development programmes has become of greater concern,^8^ as have the impacts of contextual and cultural factors that influence acceptance and uptake of faculty initiatives.^4^, ^5^ There is now a focus on harnessing the added value of social and professional networks, and the communities of practice found in the workplace.^4^, ^5^, ^8^, ^10^

Universities already implement peer support strategies in classroom settings, including in medical programmes.^11^, ^12^, ^13^ Simultaneously, research has increased our theoretical understanding of the peer observation process and identified key components and models of peer support.^13^, ^14^ Gosling's^14^ pivotal work identified three models of peer support that are, respectively, evaluative, developmental and collaborative. Evaluative and developmental models commonly engage experts to make judgements about teaching performance. Collaborative models, however, aim to promote self‐reflection and growth through non‐judgemental feedback amongst peers. Through their reliance on peers as equals, and negotiated practices, collaborative models of faculty development emphasise relationships, ‘reflective practice based on dialogue’^14^ and the ‘social enterprise’ of learning.^8^

Although a greater focus on access to peer coaching and mentoring opportunities to increase workplace‐based teaching effectiveness is supported by two Best Evidence Medical Education (BEME) guides,^5^, ^15^ the use of peers as a support strategy to improve clinical teaching is still infrequent in medical workplace contexts.^5^, ^16^

### Aims of the study

This paper presents an integrative review^17^ investigating the theoretical and empirical literature on the use of peer support strategies as educational interventions for clinical teachers in health care workplaces. The term ‘clinical teaching’ is applied here to include all activities undertaken by a health professional relating to the development of a learner in the workplace. The overarching research question was: In the health professions, is workplace‐based peer support for clinical teachers an acceptable and effective faculty development strategy?

The inquiry specifically aimed to:

1. describe and analyse existing faculty development initiatives that incorporate peer support strategies for clinical teachers in the health care workplace;
2. assess the quality of the faculty development initiatives, and
3. identify key challenges and benefits to implementing peer support strategies in order to identify knowledge gaps for future research.

## Methods

Our inquiry was underpinned by a pragmatist epistemology^18^, ^19^ in which knowledge and the research process are assumed to be situated within social, historical and political contexts, and pluralistic methods are used to uncover knowledge about problems. Across the selected studies we employed an integrative approach^17^ to extract and code data from the primary studies. We then implemented an iterative comparative analysis with ongoing refinement and validation to manage the varied data sources from the diverse methodologies employed by authors. Cook et al.^20^ support this approach as it widens the scope of studies included in the review beyond those with purely experimental designs.

### Search strategy

We used two overarching methods to identify the manuscripts: electronic database searches designed and conducted by a research librarian (author RAD), and further targeted manual searches undertaken by the remaining authors. The electronic search strategy was informed by scoping exercises in the OVID MEDLINE database, and then accurately translated for the following databases: EMBASE (OVID); PsycINFO (OVID); CINAHL (Cumulative Index to Nursing and Allied Health Literature) (EBSCOhost); Web of Science, and Informit (Health and Education subsets only). Searches comprised combinations of database‐specific subject headings, where available, and a wide range of text words to optimise search sensitivity. The search covered the years 2004–2017. Figure S1 shows a PRISMA (preferred reporting items for systematic reviews and meta‐analyses) flow chart and key search terms. Full search strategies are available on request.

The database search was supplemented with a table of contents search of six leading medical education journals (*Academic Medicine*,* Medical Education*,* Medical Teacher*,* BMC Medical Education*,* The Clinical Teacher* and *Focus on Health Professional Education*). As an additional process to ensure search strategy rigour, we included a forward citation check and manual review of the reference lists of included papers.

### Inclusion criteria

The inclusion criteria for the review required:

1. participants to be health professionals (papers involving any other profession, including higher education, were excluded), and
2. peers to be workplace colleagues or peers, with or without educational expertise (not students).

Peer support strategies were required to:

1. have been developed and conducted using an explicit process;
2. have been situated in the clinical workplace (not classroom or simulation laboratory);
3. have been aimed at improving clinical teaching or supervision activities (not clinical practice), and
4. have included any but not necessarily all of the following elements: briefing; observation; debriefing or discussion, and reflection.

Finally, papers were required to have been published in peer‐reviewed publications.

### Study records

All citations identified by electronic database searches and manual content page searching were downloaded into EndNote X8 (Clarivate Analytics, Philadelphia, PA, USA) and duplicates removed. Each of three authors (NC, HW and RLP) screened one‐third of all citations, applying the inclusion criteria to the title, abstract, keywords and publication type. A random sample of 5% of excluded papers were reviewed by all authors, which is a technique recommended by Hammick et al.^21^ This resulted in 100% agreement. We sourced the full texts of papers for which relevance could not be determined by citation alone.

### Data analysis and synthesis

Three authors independently extracted information from the included papers using a standardised data table. The key information comprised study design, context, sample size, analysis of the peer support strategy, evaluation conducted and overall conclusions. We also noted any qualitative analysis outcomes or author comments on the process. Tables S1 and S2 show the results of this process.

### Quality appraisal of included papers

In tandem with a detailed descriptive analysis and synthesis, quality was appraised using two methods. Firstly, applying Cook and Ellaway's^22^ evaluation framework, we examined the type of evaluation data reported and aligned the data to typical stages in the educational design cycle. We regard the ‘cycle’ as iterative, encompassing needs analysis, design and development of peer support strategies, evaluation of implementation processes and outcomes, and evidence of sustainability and/or wider dissemination. This framework provided a means for synthesising quantitative and qualitative data, and contextual and educational processes,^8^, ^23^, ^24^, ^25^ while still incorporating the more traditional outcomes measures emphasised by Kirkpatrick and Kirkpatrick (reaction, learning, behaviour and results).^26^ Using this first method, participants’ responses to peer support strategies were compared and contrasted, along with changes to teaching behaviour and workplace culture, and impact on student learning. A second quality appraisal was conducted using the BEME scale (1 = no clear conclusions, 5 = results are unequivocal)^21^ to rate the strength of the findings in the subgroup of papers classified as research reports.

## Results

The electronic search strategies identified 5735 citations. The removal of duplicates left 4198 citations for screening. Full‐text papers were obtained for 74 citations and an additional two papers were found via the journal contents search. Of these 76 papers, only 27 fully met the inclusion criteria. A further seven papers were identified by checking the reference lists of these included papers and by forward citation searching. Thirty‐four unique papers were therefore included in the review. The search process is shown in PRISMA flow chart format^27^ in Figure S1.

### General characteristics of included papers

Table 1 details the characteristics of the included papers.^13^, ^28^, ^29^, ^30^, ^31^, ^32^, ^33^, ^34^, ^35^, ^36^, ^37^, ^38^, ^39^, ^40^, ^41^, ^42^, ^43^, ^44^, ^45^, ^46^, ^47^, ^48^, ^49^, ^50^, ^51^, ^52^, ^53^, ^54^, ^55^, ^56^, ^57^, ^58^, ^59^, ^60^ Most of the studies had been conducted within the medical profession, in the USA, and reported on the implementation of a peer support strategy. Notably, there were no interprofessional studies. The majority of the studies were research reports using surveys, focus groups or observation to collect data from small samples. Although most of the papers described their implementation of a peer support strategy, or explained a potential strategy (‘tell how’ papers), four aimed to assess the acceptability of a peer support strategy within the workplace (‘test the waters’ papers).

**Table 1:** Key characteristics, design features and evaluation results reported in the included papers (*n* = 34)

Characteristics	Studies, *n* (%)^a^
Geographic location	
USA^28^, ^29^, ^30^, ^31^, ^32^, ^33^, ^34^, ^35^, ^36^, ^37^, ^38^, ^39^, ^40^, ^41^, ^42^, ^43^, ^44^, ^45^, ^46^, ^47^	20 (59%)
UK^13^, ^48^, ^49^, ^50^, ^51^, ^52^, ^53^, ^54^, ^55^	9 (26%)
Canada^56^, ^57^	2 (6%)
Australia^58^, ^59^	2 (6%)
Australia and UK^60^	1 (3%)
Profession	
Medicine^13^, ^28^, ^29^, ^31^, ^34^, ^36^, ^38^, ^39^, ^40^, ^41^, ^42^, ^43^, ^44^, ^45^, ^46^, ^47^, ^48^, ^50^, ^51^, ^52^, ^53^, ^54^, ^55^, ^60^	24 (71%)
Nursing^30^, ^37^, ^56^, ^58^	4 (12%)
Dentistry^49^, ^57^	2 (6%)
Pharmacy^32^, ^33^	2 (6%)
Counselling^35^	1 (3%)
Physiotherapy^59^	1 (3%)
Aim of paper	
Implement peer support strategy^29^, ^32^, ^33^, ^34^, ^35^, ^36^, ^38^, ^39^, ^40^, ^42^, ^44^, ^45^, ^46^, ^49^, ^51^, ^53^, ^54^, ^55^, ^56^, ^57^, ^58^, ^59^	22 (65%)
Tell how (to implement strategy)^13^, ^28^, ^31^, ^37^, ^41^, ^47^, ^50^, ^52^	8 (24%)
Test the waters (assess acceptability of strategy)^30^, ^43^, ^48^, ^60^	4 (12%)
Type of study	
Research report^29^, ^32^, ^34^, ^36^, ^38^, ^39^, ^42^, ^43^, ^44^, ^45^, ^46^, ^48^, ^49^, ^51^, ^53^, ^54^, ^55^, ^56^, ^57^, ^59^, ^60^	21 (62%)
Showcase^30^, ^33^, ^35^, ^37^, ^40^, ^50^, ^58^	7 (21%)
How to guide^13^, ^28^, ^31^, ^41^, ^47^, ^52^	6 (18%)
Design features of the peer support strategy	
Peer support model	
Collaborative^13^, ^28^, ^32^, ^34^, ^35^, ^38^, ^39^, ^40^, ^41^, ^45^, ^49^, ^51^, ^52^, ^55^, ^56^, ^57^, ^58^, ^59^, ^60^	19 (56%)
Developmental^29^, ^30^, ^31^, ^36^, ^42^, ^43^, ^44^, ^46^, ^47^, ^48^, ^50^, ^53^	12 (35%)
Evaluative^33^, ^37^, ^54^	3 (9%)
Strategy type	
Workplace observation^13^, ^28^, ^29^, ^30^, ^31^, ^32^, ^33^, ^34^, ^36^, ^37^, ^39^, ^41^, ^42^, ^43^, ^44^, ^45^, ^46^, ^47^, ^48^, ^49^, ^50^, ^52^, ^53^, ^54^, ^58^, ^60^	26 (76%)
Community of practice^35^, ^40^, ^51^, ^57^	4 (12%)
Reflective practice^38^, ^55^, ^56^, ^59^	4 (12%)
Nature of participation (if defined)	
Voluntary^28^, ^31^, ^32^, ^34^, ^35^, ^36^, ^38^, ^39^, ^40^, ^43^, ^48^, ^49^, ^51^, ^53^, ^54^, ^55^, ^56^, ^57^, ^58^, ^59^	20 (59%)
Mandated^29^, ^30^, ^33^, ^37^, ^44^, ^46^	6 (18%)
Status of peer (if defined)	
Clinician peer^13^, ^28^, ^30^, ^31^, ^32^, ^34^, ^35^, ^39^, ^40^, ^41^, ^42^, ^45^, ^46^, ^48^, ^49^, ^51^, ^52^, ^54^, ^55^, ^57^, ^58^	21 (62%)
Clinician educator^33^, ^36^, ^37^, ^38^, ^43^, ^44^, ^53^, ^56^, ^59^	9 (26%)
Observation guide or tool (if described/used)	
Self‐developed^29^, ^31^, ^32^, ^34^, ^36^, ^41^, ^42^, ^44^, ^45^, ^46^, ^47^, ^49^, ^50^, ^53^, ^54^	15 (44%)
Informed by professional competencies, or validated tool^28^, ^30^, ^33^, ^37^, ^39^, ^43^	6 (18%)
Process included^b^	
Training^13^, ^29^, ^30^, ^31^, ^33^, ^34^, ^35^, ^37^, ^38^, ^39^, ^41^, ^42^, ^43^, ^44^, ^46^, ^48^, ^49^, ^50^, ^51^, ^53^, ^56^, ^57^, ^59^	23 (68%)
Pre‐strategy briefing to clarify process^13^, ^30^, ^31^, ^32^, ^33^, ^34^, ^35^, ^36^, ^37^, ^38^, ^39^, ^40^, ^41^, ^43^, ^47^, ^48^, ^49^, ^50^, ^51^, ^52^, ^53^, ^56^, ^57^, ^58^	24 (71%)
Explicit mutual agreement on goals of peer support^13^, ^30^, ^34^, ^35^, ^36^, ^38^, ^40^, ^41^, ^43^, ^47^, ^48^, ^49^, ^51^, ^52^, ^53^, ^56^, ^57^	17 (50%)
Debrief/feedback immediately post‐strategy^13^, ^29^, ^31^, ^32^, ^34^, ^35^, ^36^, ^39^, ^40^, ^41^, ^42^, ^43^, ^45^, ^47^, ^49^, ^51^, ^52^, ^53^, ^54^, ^55^	20 (59%)
Explicit reflection on learning by participant^13^, ^28^, ^30^, ^31^, ^33^, ^34^, ^35^, ^36^, ^38^, ^39^, ^40^, ^41^, ^43^, ^44^, ^45^, ^47^, ^48^, ^49^, ^51^, ^52^, ^53^, ^54^, ^55^, ^56^, ^57^, ^58^, ^59^	27 (79%)
Evaluation data reported^b^, ^c^	
Design and development of peer support strategy	22 (65%)
Needs analysis^30^, ^40^, ^43^, ^48^, ^50^, ^57^, ^59^, ^60^	8 (24%)
Development process^13^, ^30^, ^31^, ^32^, ^33^, ^35^, ^37^, ^39^, ^40^, ^41^, ^43^, ^44^, ^46^, ^47^, ^50^, ^53^, ^56^, ^57^, ^59^	19 (56%)
Pilot^30^, ^44^, ^53^, ^54^	4 (12%)
Evaluation of process during implementation^28^, ^35^, ^39^, ^44^, ^56^	5 (15%)
Evaluation of outcomes	19 (56%)
Participant reaction^29^, ^36^, ^39^, ^45^, ^46^, ^49^, ^51^, ^53^, ^54^, ^55^, ^57^, ^59^ or intention to change^36^, ^39^, ^45^, ^49^, ^53^	12 (35%)
Changed teaching behaviour^34^, ^36^, ^39^, ^42^, ^45^, ^46^, ^53^, ^55^, ^56^, ^57^ or impact on student learning^32^, ^38^, ^44^, ^53^, ^59^	14 (41%)
Evaluation of sustainability and dissemination^34^, ^44^, ^49^, ^55^, ^57^	5 (15%)

a Rounding effect means percentages may not add to 100.

b Percentages are not additive in the following sections as each paper may have reported on more than one aspect.

c Based on Cook and Ellaway's evaluation framework.^22^

### Design features of the peer support strategy

Using our previous work mapping peer support strategies in the workplace,^61^ and drawing on O'Sullivan and Irby's model of faculty development,^8^ we analysed the design features of the peer support strategies reported in the papers. As Table 1 shows, over half of the papers favoured Gosling's collaborative model of peer support. The reviewed literature emphasised the trend for voluntary participation in a peer support strategy, and direct workplace observation by a clinician peer. Only one paper reported observation by videoconferencing to enable participation across dispersed sites.^29^ Although 21 studies used observation guides, the majority of which were self‐developed, only six papers detailed how this was achieved. Observations were often preceded by training in the process, and a briefing that encouraged participants to generate their own goals for the observation. The time taken to complete a peer observation varied widely (mean time: 159 minutes; range: 8 minutes to 1 day), and generally included immediate feedback from the peer observer and reflection on learning outcomes.

### Evaluation of the design and implementation

The majority of papers (*n* = 22, 65%) reported on the design and development of the peer support strategy (Table 1). Eight papers described a structured workplace needs analysis to determine the viability and acceptability of the peer support strategy, but only three of these described its subsequent implementation.^30^, ^57^, ^59^ Although 19 papers described the development of their respective programmes, only four conducted a pilot run.^30^, ^44^, ^53^, ^54^

Very few papers (*n* = 5, 15%) evaluated the study's implementation process. However, examples that were documented included personal reflections during implementation,^28^, ^35^, ^56^ modifications to enhance feedback processes following peer observation,^44^ and the identification of unanticipated outcomes such as direct benefits to the observer.^28^, ^56^

### Evaluation of outcomes: benefits and challenges for participants and the workplace

Over half of the included papers (*n* = 19, 56%) provided outcome measures that could be attributed to Kirkpatrick's four levels of training evaluation. These included 12 papers that utilised surveys or interviews to evaluate participant reactions or attitudes towards the peer support strategy, and five papers that captured participants’ intentions to change their clinical teaching practice (Table 1).

In terms of outcomes for participants, initial anxiety or misgivings^13^, ^41^, ^46^, ^49^, ^52^, ^53^, ^56^ were ameliorated by discovering that the peer support strategy was a reciprocal^13^, ^41^, ^45^, ^53^ and valuable^28^, ^30^, ^34^, ^36^, ^43^, ^44^, ^51^, ^60^ process. Intentional, focused feedback and opportunities for reflection were reported to contribute positively to a productive culture of teaching in the workplace.^13^, ^33^, ^40^, ^44^, ^55^ In turn, this reduced participants’ sense of isolation as teachers,^13^, ^49^, ^57^ aided more widespread recognition of the benefits of teaching, and increased overall commitment to teaching.^36^, ^46^, ^58^ Participants reported the process confirmed the quality of existing teaching practice^30^, ^38^, ^49^, ^56^ and provided direction to improve teaching practice.^32^, ^34^, ^35^, ^39^, ^42^, ^44^, ^45^, ^53^, ^57^, ^58^, ^60^ Growth in confidence in giving feedback was specifically noted.^31^, ^39^, ^46^

Additional impacts for participants included changes to micro‐teaching skills and behaviours. Evidence was provided via self‐reports^39^, ^46^, ^53^, ^55^, ^57^ and direct observations of change.^34^, ^36^, ^42^, ^45^, ^56^ Examples of change included enhanced questioning skills,^34^, ^36^, ^42^ better organisation of teaching,^34^, ^36^, ^45^ strategies to engage learners at multiple levels,^34^, ^36^ and heightened awareness of how to assist learners to self‐evaluate.^56^ Observers found unexpected learning benefits in watching their peers teach.^13^, ^41^, ^45^, ^53^ The impact of peer support strategies on students’ experiences of clinical teaching were less commonly reported^32^, ^38^, ^44^, ^53^, ^56^, ^59^ and all papers shied away from suggesting any links between improved student learning outcomes and implementation of improved clinical teaching practices.

In addition to benefits and challenges for participants, significant contextual workplace challenges emerged from the reported outcome evaluations. Common barriers included a lack of time for participation,^13^, ^28^, ^32^, ^33^, ^43^, ^48^, ^52^, ^55^, ^56^, ^57^ and general logistical issues.^36^, ^46^, ^55^ Three papers reportedly addressed these barriers by embedding peer support strategies into organisational routines.^34^, ^40^, ^46^ In one example participants prepared written reflections prior to peer‐facilitated group discussions, which fostered the development of a shared knowledge base, the cultivation of relationships and the sustaining of a community of practice.^40^ Additional workplace benefits reportedly included identification of further faculty development needs^44^, ^49^ and anecdotal changes to workplace culture, such as an enhanced sense of community and improved communication between clinical teachers.^55^, ^57^

Few papers provided long‐term data describing the programme's sustainability. One described multiple iterations^34^ and one documented dissemination to other health workplaces.^44^ Overall, the length of implementation varied widely from 2 weeks^42^ to 5 years^34^ (Tables S1 and S2).

### Further quality appraisal of the evidence

Overall, evaluation data were reported in all except two of the papers,^52^, ^58^ and notably none reported evaluation that spanned all stages of the educational design cycle. For the subset classified as research reports, and evaluated using the BEME strength of evidence rating (Table 1, *n* = 21), there was a wide variation in the degree to which results supported conclusions (Table S2). Two of the four papers^32^, ^42^, ^45^, ^54^ with the lowest BEME ratings (1 or 2) were short reports that lacked the detail necessary to assess the conclusions reported.^42^, ^45^ One of these reported a randomised controlled trial research design,^42^ but was limited by low participant numbers in the study group (*n* = 6). Generally, in studies in which quantitative data were collected and analysed, sample sizes were low^32^, ^39^, ^45^, ^59^ and hence any statistical findings should be read with caution.

Despite these limitations, 11 of the 21 (52%) papers were deemed to have reported conclusions based on the results collected (BEME rating 4: results clear and very likely to be true).^36^, ^38^, ^39^, ^46^, ^48^, ^49^, ^51^, ^56^, ^57^, ^59^, ^60^

## Discussion

Informed by contemporary models of faculty development,^5^, ^8^ this integrative review enabled us to draw conclusions about how peer support strategies influence, and are influenced by, the social systems and cultures operating within health care workplaces. The review is significant because it addresses a need, identified by prominent medical education researchers,^24^, ^62^, ^63^ to compare, contrast and synthesise educational studies that report on disparate methodologies, contexts and challenges.^64^ Expanding our evaluative assessment of papers beyond commonly used outcomes‐oriented methods (i.e. Kirkpatrick's four training levels) allowed us to include rich contextual information about participants and their workplace contexts and cultures in the analysis. Had we included only papers referring to outcome measures, over half of the papers would not have been included in the review (*n* = 18, 53%), thereby limiting our understanding of the impact of workplace culture and participant engagement. Similarly, if only research reports (*n* = 21) had been included, valuable data describing the design of peer support strategies would have been omitted.

**Figure 1:**
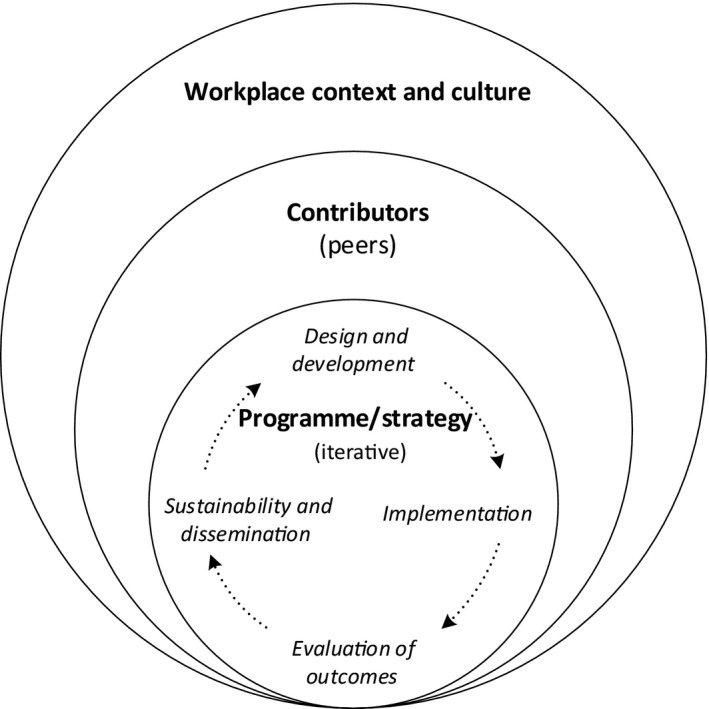
A sociocultural model of elements impacting health care workplace peer‐supported faculty development for teaching

Adapting O'Sullivan and Irby's^8^ and Steinert et al.'s^5^ models, and incorporating evidence from our review, we conceptualised workplace‐based peer support from a systems perspective (Fig. 1). We concluded that the formulating of an acceptable and effective faculty development strategy requires that three integrated factors are accounted for: the context and culture in which the programme takes place; the characteristics of the participants, whom we propose are ‘contributors’, and the educational design of the programme. This sociocultural conceptualisation frames the following discussion of key findings.

We contend that to create and manage an effective peer support strategy, acceptable to clinicians teaching in the workplace, typical educational design processes must be attended to. It is important to select design and development features appropriate to the unique workplace context, to engage in evaluation throughout (particularly process evaluation during and after implementation), to capture outcomes for participants and changes in workplace behaviour, culture and professional networks, and to generate plans that enable sustainability and dissemination beyond the participant group. These design factors impact engagement, which, in turn, is impacted by the characteristics of the participant contributors, and workplace context and culture. All the elements are interdependent and impact on one another.

### Addressing contextual and cultural challenges through design

Health care workplaces have complex cultures, and efforts to change these require time and patience.^65^ Therefore unpacking the professional culture into which peer support strategies are to be embedded may help identify barriers to engagement, and guide design choices. The inclusion of design elements such as needs assessments, pilots and preparatory briefing sessions may assist in this process of teasing out contextual, cultural and leadership issues. On this point, we identified a number of studies (referred to as ‘test the water’ papers (Table 1)), in which a needs analysis or environmental scan was conducted. Strategies reported included an initial survey to identify participant preconceptions,^60^ the adoption of grassroots involvement in the design,^30^ and orientation briefings to ensure understanding of purpose and allay misconceptions of surveillance.^43^, ^48^ Although some papers reported the development of psychometrically valid and reliable observation tools,^30^, ^32^, ^33^, ^37^, ^39^, ^43^ we suggest that workplaces could instead invest scarce time in building a culture of supportive peer relationships, which will become the foundation for successful implementation strategies.

From the reviewed papers, therefore, two design approaches emerged as particularly successful in addressing educational design issues. These were initiatives that employed a collaborative model of peer support, and/or the selection of peers based on trusted and respected relationships, rather than reliance on outside expertise. By choosing a collaborative model for peer support, the emphasis becomes one of encouraging democratic relationships between equals, facilitation of mutual learning,^14^ and being a ‘contributor’ to workplace faculty development. By comparison, the evaluative and developmental models, respectively, focus on identifying underperformance and demonstrating competency.^11^ For a collaborative model to succeed, however, participants must take responsibility for their own learning and not assume that only education experts provide feedback. Trusted peer contributors become invaluable and valid feedback providers, and reciprocity between contributors promotes personal accountability for professional development. Additionally, clinician peers are more readily available in the workplace compared with external ‘education experts’. Availability then becomes an enabler of participation.

### Benefits for contributors and the workplace

Our review casts new light on the role of O'Sullivan and Irby's ‘facilitators’,^8^ who, particularly in the preferred collaborative model, are equal contributors, rather than experts. This compares with the more passive participant role in faculty development strategies that rely on ‘outside’ education expert facilitators. A key outcome for contributors to the peer observation process was immersion in the process itself. By gathering qualitative evidence about their teaching practice, individuals created a personalised resource for ongoing professional development and reflection.^28^, ^34^, ^46^, ^49^ Personal reflection on self‐generated goals^38^, ^40^, ^45^, ^51^, ^53^, ^55^ is likely to encourage ownership of learning outcomes, which may not occur in contexts in which compliance instruments created by others are used, such as ‘tick the box’ observational tools.

Of course, attempts to change organisational culture bring risks and challenges. It is not easy to develop a culture that supports the unique conditions of the targeted workplace. However, embedding a peer‐focused programme as part of regular departmental practice can militate against opposition from teams or individuals.^43^ Finn et al.,^34^ for example, embedded their strategy as a regular workplace routine and reported the longest duration of all studies. They also noted the concomitant rapid development of novice clinical teachers. Such positive outcomes reflect a workplace culture that prioritises and normalises peer‐focused faculty development. We conclude, therefore, that by conceptualising peer support processes within a sociocultural model, the relationships between cultural values^44^ and shared teaching strategies become apparent,^36^ the positive collective effect on the organisation becomes visible,^30^, ^57^ the status of workplace teaching is elevated,^49^ and collaboration and relationship building improve.^40^, ^43^, ^49^, ^55^

With regard to sustainability, there was evidence that this building of relationships and networks within the workplace could lead to the development of self‐sustaining programmes,^57^ consistent with the features of a community of practice.^10^, ^40^, ^55^ It was also noted that participation encouraged individuals to seek out further teaching improvement opportunities.^36^ Hence embedding a peer support strategy within a wider programme of faculty development may inspire engagement by late adopters, following the example set by their colleagues.

### Future research

As a result of this integrative review, and in response to our final research question, we identified a number of gaps in the literature and areas for future investigation. In particular, although researchers highlighted the need to illuminate factors impacting the implementation of educational interventions,^21^, ^24^, ^25^, ^66^ few systematically collected or rigorously analysed process evaluation data. We agree with other researchers^5^, ^20^, ^23^ that clarification studies asking the ‘how’ and ‘why’ questions should be a key research focus. These investigations might explore and evaluate alternative design characteristics to address issues such as access (e.g. the efficacy of synchronous and asynchronous digital communications) and peer availability. Peer support faculty development programmes would benefit from better understanding of the roles of social, cultural, professional and interprofessional networks in promoting workplace clinical teaching excellence,^67^ and of the mechanisms required to sustain programmes. Additionally, gathering evidence of changed teaching practice and improved student learning outcomes associated with programmes would be valuable.

Future studies could be framed around conceptual models of faculty development, including our sociocultural model, and those offered by O'Sullivan and Irby,^8^ and Steinert et al.^5^ Additionally, education design research, or design‐based research, shows promise as a worthwhile research paradigm for medical education research,^68^, ^69^, ^70^ and faculty development research,^71^ as it blends design, research and practice.^72^

### Limitations

A factor limiting this review was the difficulty of managing the critical appraisal of a heterogeneous set of papers. Methodological design flaws in the papers compounded the difficulty. These included the frequent omission of any reporting of the study's underlying education philosophy, the use of ambiguous research questions, opportunistic data collection, and incomplete reporting of data analysis processes. To minimise the impact of these limitations, we applied consistent and structured iterative comparative techniques during the final selection of articles, the extraction and coding of data, and the analysis and synthesis of results. One other limitation was the restriction of our search strategy to English‐language papers published during 2004–2017, which narrowed the reach of the review. This was, however, compensated for by careful consideration of other faculty development review papers reporting earlier studies.^4^, ^5^, ^15^

## Conclusion

This integrative review provides an evidence‐based perspective on strategies used to promote peer‐supported faculty development of teaching practice in health care‐related workplace settings. Positioning the evidence within a sociocultural model, our findings confirm the acceptability and effectiveness of faculty development strategies that focus on peer support as a means for improving teaching and learning practice in the workplace. Those initiatives that adopted collaborative and consultative approaches, and utilised trusted peer contributors, rather than outside experts, appeared to be most acceptable to participants and most effective in practice.

Using our sociocultural model, we suggest that strategy development and implementation must acknowledge and account for: (i) educational design elements; (ii) workplace relationships and the concerns of contributors, and (iii) workplace context and culture. By carefully designing workplace‐based peer support programmes around an iterative educational design process, and encouraging voluntary participation with trusted colleagues, reciprocal benefits for collaborators can be realised. Importantly, by fostering the beneficial outcome of mutual reflection on clinical teaching practice, positive relationships, communities and connections can be built amongst clinical teachers in the workplace.

## Contributors

NC, HW and RLP contributed to the conception and design of the work, the analysis and interpretation of data, and the drafting and critical revision of the paper. RAD contributed to the design of the work, and the drafting and critical revision of the paper. All authors approved the final manuscript for publication and have agreed to be accountable for all aspects of the work, including the investigation and resolution of questions related to its accuracy or integrity.

## Funding

none.

## Conflicts of interest

none.

## Ethical approval

not applicable.

## Supporting information


**Figure S1**. PRISMA flowchart including key search terms and search strategy.

**Table S1**. Descriptive data extracted from the included papers.

**Table S2**. Further details of the results reported in Table 1 for each element of the evaluation‐based framework.
