# Supplementary material for: Peer‐supported faculty development and workplace teaching: an integrative review
Source: Med Educ. 2019 Jun 25;53(10):978–88. doi: 10.1111/medu.13896 (PMC6771963; doi:10.1111/medu.13896)
Supplement: Supplementary file 1 — Figure S1. PRISMA flowchart including key search terms and search strategy. [file MEDU-53-978-s001.pdf]

Supplementary Material
PRISMA flowchart including key search terms and search strategy

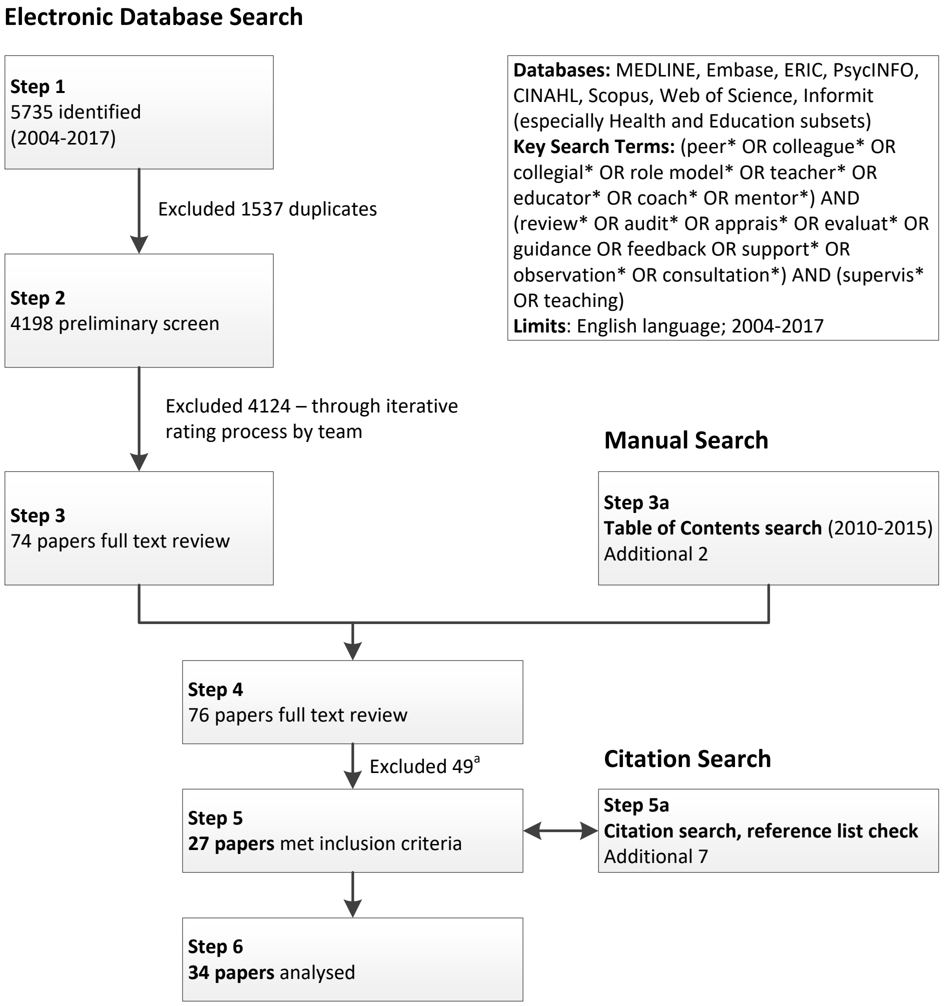

[a]Exclusion reasons: No explicit process 15; Not in clinical workplace 18; Not health professionals 2; Focussed on clinical practice rather than improving teaching 10; Peers not used 4
