# Supplementary material for: Peer‐supported faculty development and workplace teaching: an integrative review
Source: Med Educ. 2019 Jun 25;53(10):978–88. doi: 10.1111/medu.13896 (PMC6771963; doi:10.1111/medu.13896)
Supplement: Supplementary file 2 — Table S1. Descriptive data extracted from the included papers. [file MEDU-53-978-s002.docx]

Supplementary Material

*Table S1: Descriptive data table of included papers*

| **Author (Year)** | **Profession (Country)**  **Aim of paper**  **Method**  **Sample size (n)** | **Model**  **Peer type (selection process)**  **Participation** | **Strategy**  **Components included^a^**  **Observation guide**  **Timing of feedback** | **Time per participant**  **Length of program** | **Qualitative findings:**  **Emotional response**  **Value of process to observer** | **Qualitative findings:**  **Influence on success**  **Constraints to ongoing use** |
| --- | --- | --- | --- | --- | --- | --- |
| Adshead L, White PT, Stephenson A. (2006)^48^ | Medicine (UK)  Test the waters  Mixed methods – Surveys and focus group  n >300 | Developmental  Clinician peer (self-selected)  Voluntary | Workplace observation cycle  OrBrObFR  Guide not applicable  Feedback provided later | NR^b^  NR | Fear of scrutiny  Preference for being observed rather than observer | Addressing of participant reservation  Time |
| Barnard A, Harvey T, Theobald K, Tippett V, Rider T. (2016)^58^ | Nursing (Australia)  Implement peer support strategy  Program description  n= NR | Collaborative  Clinician peer (NR)  Voluntary | Workplace observation cycle  BrObFR  NR  NR | NR  NR | NR  NR | NR  NR |
| Beckman TJ. (2004)^28^ | Medicine (USA)  Tell how  Literature and expert opinion  n <20 | Collaborative  Clinician peer (NR)  Voluntary | Workplace observation cycle  ObFR  Guide informed by professional competencies or validated tool  Feedback provided later | NR  6 months | NR  High | NR  Time |
| Biery N, Bond W, Smith AB, Leclair M, Foster E. (2015)^29^ | Medicine (USA)  Implement peer support strategy  Qual - Survey  n <20 | Developmental  Peer not defined (allocated)  Voluntary | Workplace observation cycle  ObFR  Self-developed observation guide  Immediate feedback | 8-60 mins (av 21 mins)  13 months | NR  NR | Commitment to a process  NR |
| Blauvelt MJ, Erickson CL, Davenport NC, Spath ML. (2012)^30^ | Nursing (USA)  Test the waters  Program description  n <20 | Developmental  Clinician peer (self-selected)  Voluntary | Workplace observation cycle  OrBrObFR  Guide informed by professional competencies or validated tool  Feedback provided later | 1-3 hours  NR | Received positively  NR | Workplace commitment  NR |
| Cairns AM, Bissell V, Bovill C. (2013)^49^ | Dentistry (UK)  Implement peer support strategy  Qual - Interviews  n <20 | Collaborative  Clinician peer (allocated)  Voluntary | Workplace observation cycle  OrBrObFR  Self-developed observation guide  Immediate feedback | 6 hours  ‘One round’ | Initially reserved but reassured by outcome  High | Workplace commitment  None |
| Caygill R, Peardon M, Waite C, McIntyre I, Bradley D, Wright J. (2017)^60^ | Medicine (Australia and UK)  Test the waters  Qual - Survey  n>300 | Collaborative  Peer not defined (NR)  Voluntary | Workplace observation cycle  Components not specified  NR | NR  NR | Received positively  NR | NR  NR |
| Chandler D, Snydman L, Rencic J. (2009)^31^ | Medicine (USA)  Tell how  Literature and expert opinion | Developmental  Clinician peer (allocated)  Voluntary | Workplace observation cycle  OrBrObFR  Self-developed guide  Immediate feedback | NR  3 years | NR  NR | Workplace commitment; trust  NR |
| Cox CD, Peeters MJ, Stanford BL, Seifert CF. (2013)^32^ | Pharmacy (USA)  Implement peer support strategy  Mixed methods - observation data and survey  n <20 | Collaborative  Clinician peer (self-selected)  Voluntary | Workplace observation cycle  BrObF  Self-developed guide  Immediate feedback | One day  ‘One round’ | NR  NR | Workplace commitment  Time |
| Elmore L, Blair M, Edgerton L. (2014)^33^ | Pharmacy (USA)  Implement peer support strategy  Program description  n 21-50 | Evaluative  Clinician educator (allocated)  Voluntary | Community of practice  OrBrObR  Guide informed by professional competencies or validated tool  NR | NR  2 years | NR  NR | Workplace commitment, using a process  Time |
| Finn K, Chiappa V, Puig A, Hunt DP. (2011)^34^ | Medicine (USA)  Implement peer support strategy  Qual - Description  n <20 | Collaborative  Clinician peer (allocated)  Voluntary | Workplace observation cycle  BrObFR  Self-developed guide  Immediate feedback | 2 hours  5 years | NR  High | Participant commitment  NR |
| Fry H, Morris C. (2004)^50^ | Medicine (UK)  Tell how  Program description  NR | Developmental  Peer not defined (NR)  Voluntary | Workplace observation cycle  OrBrOb  Self-developed guide  NR | NR  NR | NR  NR | NR  NR |
| Granello DH, Kindsvatter A, Granello PF, Underfer-Babalis J, Moorhead HJH. (2008)^35^ | Counselling (USA)  Implement peer support strategy  Program description  n <20 | Collaborative  Clinician peer (allocated)  Voluntary | Community of practice  BrFR  Guide not applicable  Immediate feedback | Several meetings/year  NR | Initially reserved but reassured by outcome  Not applicable | Respect, recognition of power  NR |
| Gusic M, Hageman H, Zenni E. (2013)^36^ | Medicine (USA)  Implement peer support strategy  Mixed methods - observation and interview  n <20 | Developmental  Clinician educator (allocated)  Voluntary | Workplace observation cycle  BrObFR  Self-developed guide  Immediate feedback | NR  NR | Received positively  NR | Commitment to a process  Logistical challenges |
| Lundeen JD, Warr RJ, Cortes CG, Wallis F, Coleman JJ. (2015)^37^ | Nursing (USA)  Tell how  Program description  n 21-50 | Evaluative  Clinician educator (allocated)  Voluntary | Workplace observation cycle  Ob  Guide informed by professional competencies or validated tool  NR | NR  1 years | NR  NR | NR  NR |
| Mahara MS, Jones JA. (2005)^56^ | Nursing (Canada)  Implement peer support strategy  Qual - Case study  n <20 | Collaborative  Clinician educator (self-selected)  Voluntary | Reflective practice  OrBrFR  Guide not applicable  Feedback provided at multiple times | NR  1 semester | Initially reserved but reassured by outcome  Not applicable | Trust, respect  Time |
| Mai CL, Baker K. (2017)^38^ | Medicine (USA)  Implement peer support strategy  Qual – Case study  n <20 | Collaborative  Clinician educator (self-selected)  Voluntary | Reflective practice  BrFR  Guide not applicable  Feedback provided at multiple times | NR  2 years | NR  Not applicable | Trust  NR |
| Main P, Curtis A, Pitts J, Irish B. (2009)^51^ | Medicine (UK)  Implement peer support strategy  Qual - Interview  Sample <20 | Collaborative  Clinician peer (allocated)  Voluntary | Community of practice  OrBrFR  Guide not applicable  Immediate feedback | NR  NR | Received positively  Not applicable | Participant commitment, respect  NR |
| Metcalfe MJ, Farrant M, Farrant J. (2010)^52^ | Medicine (UK)  Tell how  Literature and expert opinion | Collaborative  Clinician peer (NR)  Voluntary | Workplace observation cycle  OrBrObFR  Guide not applicable  Immediate feedback | ‘Significant’  NR | Initially reserved but reassured by outcome  NR | Workplace commitment  Time |
| Mookherjee S, Monash B, Wentworth KL, Sharpe BA. (2014)^39^ | Medicine (USA)  Implement peer support strategy  Survey  n 21-50 | Collaborative  Clinician peer (allocated)  Voluntary | Workplace observation cycle  OrBrObFR  Guide informed by professional competencies or validated tool  Immediate feedback | NR  I year | Neutral  NR | Workplace commitment  NR |
| Murray SB, Levy M, Lord J, McLaren K. (2013)^40^ | Medicine (USA)  Implement peer support strategy  Program description  NR | Collaborative  Clinician peer (self-selected)  Voluntary | Community of practice  BrFR  Guide not applicable  Immediate feedback | NR  NR | NR  Not applicable | Trust  NR |
| Newman L, Roberts D, Schwartzstein R. (2012)^41^ | Medicine (USA)  Tell how  Literature and expert opinion | Collaborative  Clinician peer (self-selected)  Voluntary | Workplace observation cycle  BrObFR  Self-developed guide  Immediate feedback | NR  NR | Initially reserved but reassured by outcome  Value for both observer and observed | NR  NR |
| Parrott S, Dobbie A, Chumley H. (2006)^42^ | Medicine (USA)  Implement peer support strategy  Randomised control trial  n <20 | Developmental  Clinician peer (allocated)  Voluntary | Workplace observation cycle  OrObF  Self-developed guide  Immediate feedback | NR  2 weeks | NR  NR | NR  NR |
| Pattison AT, Sherwood M, Lumsden CJ, Gale A, Markides M. (2012)^53^ | Medicine (UK)  Implement peer support strategy  Mixed methods -survey and focus groups  n 21-50 | Developmental  Clinician educator (NR)  Voluntary | Workplace observation cycle  OrBrObFR  Self-developed guide  Immediate feedback | 2 ½ hours  2 years | Initially reserved but reassured by outcome  Value for both observer and observed | Trust  NR |
| Peyre SE, Frankl SE, Thorndike M, Breen EM. (2011)^43^ | Medicine (USA)  Test the waters  Survey  n 21-50 | Developmental  Clinician educator (NR)  Voluntary | Workplace observation cycle  BrObFR  Guide informed by professional competencies or validated tool  Immediate feedback | 90 mins-2 hours  NR | Received positively  Preference for being observed rather than observer | Trust  Time |
| Regan-Smith M, Hirschmann K, Lobst W. (2007)^44^ | Medicine (USA)  Implement peer support strategy  Mixed methods - participant–observer ethnographic analysis  n 21-50 | Developmental  Clinician educator (NR)  Voluntary | Workplace observation cycle  OrObFR  Self-developed guide  Feedback provided later | 60-90 mins (av 94)  2 years | Received positively  Not applicable | Using a learner-centred process  NR |
| Rendon P, Rao D, Pierce JR. (2015)^45^ | Medicine (USA)  Implement peer support strategy  Mixed methods - observation and survey  n <20 | Collaborative  Clinician peer (self-selected)  Voluntary | Workplace observation cycle  ObFR  Self-developed guide  Immediate feedback | 60-180 mins, av 91  9 months | NR  Value for both observer and observed | NR  NR |
| Sneddon A, MacVicar R (2016)^55^ | Medicine (UK)  Implement peer support strategy  Qual - focus group and written reflection  n 21-50 | Collaborative  Clinician educator (NR)  Voluntary | Reflective practice  BrFR  Guide not applicable  Immediate feedback | NR  NR | Initially reserved but reassured by outcome  Not applicable | Respect, using a process  Time |
| Snydman L, Chandler D, Rencic J, Sung YC. (2013)^46^ | Medicine (USA)  Implement peer support strategy  Quant- survey  n 21-50 | Developmental  Clinician peer (allocated)  Voluntary | Workplace observation cycle  OrBrObF  Self-developed guide  Feedback provided later | NR  10 months | Initially reserved but reassured by outcome  NR | Workplace commitment  NR |
| Spicer J, Torry R. (2011)^54^ | Medicine (UK)  Implement peer support strategy  Qual – survey and observation records  n <20 | Evaluative  Clinician peer (allocated)  Voluntary | Workplace observation cycle  ObFR  Self-developed guide  Immediate feedback | 1-1½ hours  NR | NR  NR | Sufficient time  NR |
| Tax CL, Doucette H, Neish NR, Maillet JP. (2012)^57^ | Dentistry (Canada)  Implement peer support strategy  Qual - survey  n <20 | Collaborative  Clinician peer (NR)  Voluntary | Community of practice  OrBR  Guide not applicable  NR | NR  1 year | NR  Not applicable | Participant commitment  Time |
| Thampy H, Kersey N. (2015)^13^ | Medicine (UK)  Tell how  Literature and expert opinion | Collaborative  Clinician peer (NR)  Voluntary | Workplace observation cycle  BrObFR  NR  Immediate feedback | NR  NR | Initially reserved but reassured by outcome  Value for both observer and observed | Trust  Time |
| Thomson K, Nguyen M, Leithhead I. (2016)^59^ | Physiotherapy (Australia)  Implement peer support strategy  Mixed methods - survey  n <20 | Collaborative  Clinician educator (allocated)  Voluntary | Reflective practice  OrR  Guide not applicable  Feedback monthly | NR  10 months | NR  Not applicable | Informal process  NR |
| Zenni E, Hageman H, Hafler J, Gusic M. (2011)^47^ | Medicine (USA)  Tell how  Literature and expert opinion | Developmental  Peer not defined (NR)  Voluntary | Workplace observation cycle  BrObFR  Self-developed guide  Immediate feedback | Minimum 30 minutes  NR | NR  NR | Respect and trust  NR |
