# Supplementary material for: Peer‐supported faculty development and workplace teaching: an integrative review
Source: Med Educ. 2019 Jun 25;53(10):978–88. doi: 10.1111/medu.13896 (PMC6771963; doi:10.1111/medu.13896)
Supplement: Supplementary file 3 — Table S2. Further details of the results reported in Table 1 for each element of the evaluation‐based framework. [file MEDU-53-978-s003.docx]

Supplementary Material

*Table S2: Further details of the results reported in Table 1 for each element of the evaluation-based framework*

| **Author (Year)** | **Type of Paper** | 1. **Design related data**   **1a:** **Contextual needs analysis and environmental scan**  **1b:** **Development process**  **1c: Pilot** | 1. **Process evaluation** | 1. **Outcomes evaluation**   **3a:** **Kirkpatrick’s Level 1-2: Reaction or intention to change teaching behaviour**  **3b: Kirkpatrick’s Level 3-4 Changed teaching behaviour or impact on student learning** | 1. **Sustainability or Dissemination**   **Workplace or organisational changes, sustainability or wider adoption** | **Strength of findings** a |
| --- | --- | --- | --- | --- | --- | --- |
| Adshead L, White PT, Stephenson A. (2006)^48^ | Research report | Focus group designed a workplace survey (1a) |  |  |  | 4 |
| Barnard A, Harvey T, Theobald K, Tippett V, Rider T. (2016)^58^ | Showcase |  |  |  |  | N/A |
| Beckman TJ. (2004)^28^ | How to guide |  | Personal reflections (2) |  |  | N/A |
| Biery N, Bond W, Smith AB, Leclair M, Foster E. (2015)^29^ | Research report |  |  | Surveying of participants to establish reactions to the observation process (3a) |  | 3 |
| Blauvelt MJ, Erickson CL, Davenport NC, Spath ML. (2012)^30^ | Showcase | Committee informed by literature reviewed workplace context (1a); leading to development of peer support strategy and observation tool (1b). Small pilot (n=3) with survey used to modify design of peer support strategy (1c) |  |  |  | N/A |
| Cairns AM, Bissell V, Bovill C. (2013)^49^ | Research report |  |  | Interviews with participants to establish reactions to the observation process and identify intentions to modify teaching practices (3a) | Help to identify further faculty development needs and options for wider adoption (4) | 4 |
| Caygill R, Peardon M, Waite C, McIntyre I, Bradley D, Wright J. (2017)^60^ | Research report | Literature and workplace survey (1a) |  |  |  | 4 |
| Chandler D, Snydman L, Rencic J. (2009)^31^ | How to guide | Personal experience informed peer support strategy (1b) |  |  |  | N/A |
| Cox CD, Peeters MJ, Stanford BL, Seifert CF. (2013)^32^ | Research report | Observation tool developed and peer support strategy negotiated with faculty (1b) |  | Compared student ratings of teaching 7 years after peer support strategy initially implemented (3b) |  | 2 |
| Elmore L, Blair M, Edgerton L. (2014)^33^ | Showcase | Professional regulations informed development of peer support strategy and observation tool (1b) |  |  |  | N/A |
| Finn K, Chiappa V, Puig A, Hunt DP. (2011)^34^ | Research report |  |  | Observation data sheet analysis showing improvements of teaching practices (3b) | Reported longitudinal sustainability of peer support strategy (4) | 3 |
| Fry H, Morris C. (2004)^50^ | Showcase | Consultation with staff (1a); personal experience informed peer support strategy and observation tool (1b) |  |  |  | N/A |
| Granello DH, Kindsvatter A, Granello PF, Underfer-Babalis J, Moorhead HJH. (2008)^35^ | Showcase | Literature and personal experience informed design (1b) | Personal reflections (2) |  |  | N/A |
| Gusic M, Hageman H, Zenni E. (2013)^36^ | Research report |  |  | Interviews with participants to establish reactions to the observation process and identify intentions to modify teaching practices (3a) with follow-up interviews about actual changes to teaching (3b) |  | 4 |
| Lundeen JD, Warr RJ, Cortes CG, Wallis F, Coleman JJ. (2015)^37^ | Showcase | Professional regulations informed development of peer support strategy and observation tool (1b) |  |  |  | N/A |
| Mahara MS, Jones JA. (2005)^56^ | Research report | Participatory research contributed to evolution of peers support strategy (1b) | Thematic analysis of documents, audio recordings, reflective statements (2) | Ethnographic analysis demonstrating changes to teaching practice and improvements in student actions (3b) |  | 4 |
| Mai CL, Baker K. (2017)^38^ | Research report |  |  | Compared student ratings of teaching between participants who completed peer observation and those who did not participate (3b) |  | 3 |
| Main P, Curtis A, Pitts J, Irish B. (2009)^51^ | Research report |  |  | Interviews with participants to establish reactions to the peer support strategy (3a) |  | 4 |
| Metcalfe MJ, Farrant M, Farrant J. (2010)^52^ | How to guide |  |  |  |  | N/A |
| Mookherjee S, Monash B, Wentworth KL, Sharpe BA. (2014)^39^ | Research report | Literature informed design of peer support strategy; Delphi process used to modify Stanford observational tool (1b) | Participation levels documented (2) | Surveying of participants to establish reactions to the observation process, and retrospective perceptions about improvements in their knowledge and teaching practices (3a); survey with self-reporting of changes to teaching practice (3b) |  | 4 |
| Murray SB, Levy M, Lord J, McLaren K. (2013)^40^ | Showcase | Need identified from mentoring meetings (1a); personal experience informed peer support strategy (1b) |  |  |  | N/A |
| Newman L, Roberts D, Schwartzstein R. (2012)^41^ | How to guide | Personal experience informed peer support strategy (1b) |  | Surveying of participants to establish reactions to the observation process (3a) |  | N/A |
| Parrott S, Dobbie A, Chumley H. (2006)^42^ | Research report |  |  | Compared observations of teaching behaviour between participants who completed peer observation and those who did not participate (3b) |  | 1 |
| Pattison AT, Sherwood M, Lumsden CJ, Gale A, Markides M. (2012)^53^ | Research Report | Literature informed design of peer support strategy and observation tool (1b). Pilot (n=3) led to inclusion of group debriefing session (1c) |  | Group debrief to establish reactions to the observation process, and survey to gather retrospective perceptions about changes to teaching practices (3a); survey with self-report of changes to teaching practice and student evaluations of teaching (3b) |  | 3 |
| Peyre SE, Frankl SE, Thorndike M, Breen EM. (2011)^43^ | Research report | Literature and workplace survey (1a) informed design of peer support strategy and observation tool development (1b) |  |  |  | 3 |
| Regan-Smith M, Hirschmann K, Lobst W. (2007)^44^ | Research report | Educational experts used preliminary observations to design peer support strategy and observation tool (1b). Pilot (n=5) used to improve feedback mechanism (1c) | Changes to peer support strategy documented and observation tool data thematically analysed (2) | Compared student ratings of teaching between participants who completed peer observation and those who did not participate (3b) | Reported wider adoption across organisation and noted organisational culture where staff requested participation in the peer support strategy (4) | 3 |
| Rendon P, Rao D, Pierce JR. (2015)^45^ | Research report |  |  | Surveying of participants to establish reactions to the observation process and identify intentions to modify teaching practices (3a); actual changes to teaching practices noted (3b) |  | 2 |
| Sneddon A, MacVicar R (2016)^55^ | Research report |  |  | Written reflections and focus group of participants about peer appraisal process (3a); self-reports of impact of peer appraisal on teaching practice (3b) | Reports of impact of peer appraisal process on workplace relationships and sense of community with their peers (4). | 3 |
| Snydman L, Chandler D, Rencic J, Sung YC. (2013)^46^ | Research report | Literature informed design of peer support strategy and observation tool (1b) |  | Surveying of participants to establish reactions to the observation process (3a); survey with self-report of changes to teaching practice (3b) |  | 4 |
| Spicer J, Torry R. (2011)^54^ | Research report | Pilot (n=8) highlighted limitations of evaluative model and led to inclusion of learner evaluations in peers support strategy (1c) |  | Anecdotal comments about the observation process (3a) |  | 1 |
| Tax CL, Doucette H, Neish NR, Maillet JP. (2012)^57^ | Research report | Meeting with potential participants (1a); literature informed development of peer support strategy (1b) |  | Surveying of participants to establish reactions to the peer support strategy (3a); survey with self-report of changes to teaching practice (3b) | Reported improvements in workplace communication between participants (4) | 4 |
| Thampy H, Kersey N. (2015)^13^ | How to guide | Literature informed development of peer support strategy (1b) |  |  |  | N/A |
| Thomson K, Nguyen M, Leithhead I. (2016)^59^ | Research report | Interview with potential participants (1a) informed design of peer support strategy (1b) |  | Surveying of participants to establish reactions to the peer support strategy (3a); student evaluations about quality of teaching (3b) |  | 4 |
| Zenni E, Hageman H, Hafler J, Gusic M. (2011)^47^ | How to guide | Personal experience informed peer support strategy (1b) |  |  |  | N/A |
